# Conserved Nuclear Localization Signal in NS2 Protein of Bombyx Mori Bidensovirus: A Potential Invertebrate ssDNA Virus Trait

**DOI:** 10.3390/v17010071

**Published:** 2025-01-06

**Authors:** Qian Yu, Jiaxin Yan, Ying Chen, Jinfeng Zhang, Qi Tang, Feifei Zhu, Lindan Sun, Shangshang Ma, Xiaoyong Liu, Keping Chen, Qin Yao

**Affiliations:** School of Life Sciences, Jiangsu University, Zhenjiang 212013, China; yanjiaxin9899@163.com (J.Y.); 3224501003@stmail.ujs.edu.cn (Y.C.); 15689321131@163.com (J.Z.); tangqi1224@163.com (Q.T.); feifzhu@ujs.edu.cn (F.Z.); sunlindan@ujs.edu.cn (L.S.); mashang@ujs.edu.cn (S.M.); liuxiaoyong@ujs.edu.cn (X.L.); kpchen@ujs.edu.cn (K.C.); yaoqin@ujs.edu.cn (Q.Y.)

**Keywords:** Bombyx mori bidensovirus, NS2, nuclear location signals (NLSs), invertebrate ssDNA virus

## Abstract

Bombyx mori bidensovirus (BmBDV), a significant pathogen in the sericulture industry, holds a unique taxonomic position due to its distinct segmented single-stranded DNA (ssDNA) genome and the presence of a self-encoding DNA polymerase. However, the functions of viral non-structural proteins, such as NS2, remain unknown. This protein is hypothesized to play a role in viral replication and pathogenesis. To investigate its structure and function, we employed phylogenetic analysis, subcellular localization, mutational analysis, and a dual-luciferase reporter system to characterize the nuclear localization signal (NLS) within NS2 and its effect on viral promoter activity. Additionally, co-immunoprecipitation and mass spectrometry were utilized to identify host proteins interacting with NS2. We identified a functional bipartite NLS in NS2, validated the combination pattern of key amino acids, and demonstrated its role in regulating viral promoter activity. Furthermore, we identified potential NLSs in NS2 homologs in other invertebrate ssDNA viruses based on sequence analysis. We also revealed interactions between NS2 and host nuclear transport proteins, suggesting that it plays a role in nuclear transport and viral replication. This research underscores the importance of NS2’s NLS in BmBDV’s life cycle and its potential conservation across invertebrate ssDNA viruses, providing insights into virus–host interactions and avenues for antiviral strategy development.

## 1. Introduction

Bombyx mori bidensovirus (BmBDV) is the only member of the genus *Bidensovirus* in the family *Bidnaviridae* that specifically infects the columnar epithelial cells of the silkworm midgut, and is one of the most seriously harmful pathogens in the sericulture industry [1]. The virus particle, similar to densoviruses, is a non-enveloped icosahedral structure with a diameter of 20–24 nm. The unique viral genome contains two linear single-stranded DNA (ssDNA) molecules, VD1 and VD2, both of which contain terminal reverse repeats and have a common sequence of 53 nts, and the terminal sequences of VD1 and VD2 can form a “pot-like” secondary structure [2]. VD1 contains four open reading frames encoding the non-structural proteins NS1, NS2, structural protein VP, and DNA polymerase PolB. VD2 contains two open reading frames encoding the non-structural protein NS3 and the structural protein P133. Krupovic reported the evolutionary scenario for the origin of BmBDV, providing a clue for the “multi origin” of this virus. Viral proteins may be evolved from various ancestors [3]. However, the evolutionary information on NS2 is still limited. The NS2 protein exists in all invertebrate ssDNA viruses with low sequence conservation. Li reported that BmBDV NS1 and NS2 have separate transcripts that are derived from overlapping promoters, P5 and P5.5 [4], which is similar to the members of the *Iteradensovirus* family [5]. This suggests that there may be differences in the expression phase and function between these two non-structural proteins. Previous studies have reported that the non-structural protein NS1 of BmBDV is homologous to the invertebrate parvoviruses NS1 and may have helicase activity, regulate ATPase activity and virulence through phosphorylation, and play an important role in the replication process of the virus [6]. The coding frame of NS2 protein is mostly contained within the coding frame of NS1, which suggests a potential overlap in their expression regulation [4]. While the position of a protein’s ORF does not directly determine its function, it can provide clues about the mechanisms of its regulation and expression. For instance, overlapping ORFs may indicate shared regulatory elements or differential splicing events that could influence the timing and levels of protein expression, thereby affecting their functional roles within the viral life cycle.

The nuclear localization of viral proteins is a critical aspect of viral replication and pathogenesis, as evidenced by recent research. Single-stranded DNA viruses are, in general, intranuclear for all or most of their replication and assembly. They often require the host cells to be undergoing nuclear transcription in the S-phase (i.e., rapidly dividing) to replicate. Both of these facts suggest that there should be strong, nuclear location signals or sequences (NLSs) [7] associated with the viral proteins to allow their entry through the nuclear pore complex to be functional. In the early steps of infection, the NLS assists in the translocation of viral genomes to the nucleus, whereas in the later steps of infection, the NLS is required for the nuclear transport of viral capsid proteins [8]. The classic NLSs (cNLSs) can be either monopartite with more than four basic amino acids or bipartite, which consists of two groups of basic amino acids together [9]. These stretches of basic amino acids then bind to β importin or α-β complexed importins to transport the protein through the nuclear pore complex into the nucleus. Adeno-associated virus (AAV) capsid proteins, for instance, have been shown to interact with importin alpha proteins via a bipartite NLS, facilitating their entry into the nucleus, which is crucial for gene therapy applications [10]. Similarly, the NS5 protein of Zikavirus (ZIKV), which contains NLSs and nuclear export signals (NESs), is found in the nuclei of infected cells and implicated in genome translation and replication [11]. The nuclear import of ZIKV NS5 is mediated by importin–alpha/beta, while its export is mediated by nuclear export protein CRM-1, highlighting the significance of nucleo-cytoplasmic transport in flavivirus replication. Barmah Forest virus (BFV) research has identified an NLS in its nsP3 protein. Mutations disrupting this NLS lead to attenuated BFV replication, suggesting that nsP3 nuclear localization may be associated with interferon antagonism and is important for viral replication and pathogenicity [12].

When BmBDV was first reported, it belonged to the genus *Densovirus* because the symptoms of virus infection in silkworms were highly similar to those of other densoviruses, and its genome was also composed of ssDNA structure. Even though this virus is currently classified in a new virus family, it still shares many similarities with other invertebrate ssDNA viruses, including the composition and function of viral proteins. However, there are limited reports on the non-structural protein function of invertebrate ssDNA viruses, and the lack of suitable sensitive cell lines to rescue viruses has also become an obstacle to in vitro studies of viral protein function. Given the intranuclear replication and assembly characteristics of ssDNA viruses, it is hypothesized that BmBDV non-structural protein NS2, like many viral proteins involved in these processes, may contain an NLS. Our interest in NS2 stems from its potential role in nuclear import, which could be pivotal for BmBDV replication and pathogenesis. Therefore, we aim to explore the cellular localization of NS2 at the cellular level and compare it with NS2 proteins of other invertebrate ssDNA viruses, providing some ideas for further elucidating the function of this protein in the future.

In this report, a phylogenetic analysis showed that the NS2 of invertebrate ssDNA viruses was close to the current classification in evolutionary branches, despite the low sequence conservation between genus. We demonstrated that the NS2 of BmBDV contained a functional bipartite NLS at its C-terminal. Mutagenesis of basic amino acids was used to identify the critical amino acids within the NLS. A bioinformatical search for all invertebrate ssDNA viruses’ NS2 was also undertaken to look for potential NLSs. The results showed that the NS2 proteins of each virus contain either a cNLS or non-classic NLSs (ncNLS) domain, and their sequence and location are relatively conserved within the same genus. Therefore, we speculate that the NS2 protein in invertebrate ssDNA viruses may have a functional conservation feature. We also analyzed the effect of the NS2 protein on viral promoter activity using a dual-luciferase reporter system. At the same time, using the recombinant Bombyx mori nuclear polyhedroviruses (BmNPV), we attempted to search for the host-interacting protein of NS2 in the midgut of silkworms.

## 2. Materials and Methods

### 2.1. Bioinformatics Software

The main bioinformatics software and websites used in this study were NCBI (https://www.ncbi.nlm.nih.gov, accessed on 30 June 2024) for obtaining non-structural proteins of invertebrate parvovirus; MEGA7 for multi-sequence comparison and construction of evolutionary trees, downloaded at https://www.megasoftware.net/ (accessed on 11 May 2024); and NLS Mapper, which predicts invertebrate parvoviruses, which can be found at https://nls-mapper.iab.keio.ac.jp/cgi-bin/NLS_Mapper_form.cgi (cNLS Mapper extracts putative NLS sequences with a score equal to or more than the selected cut-off score. Higher scores indicate stronger NLS activities. Briefly, a score of 8, 9, or 10 indicates exclusive localization to the nucleus; a score of 7 or 8 indicates partial localization to the nucleus; a score of 3, 4, or 5 indicate localization to both the nucleus and the cytoplasm; and a score of 1 or 2 indicate localization to the cytoplasm).

### 2.2. Plasmid Construction and Cell Culture

Ecoli.Fast-T1 competent cells were purchased from Vazyme (Nanjing, China), and pFastBac-Pie1-eGFP and PIBV5-mCherry were built and preserved by our lab. The fluorescence-reporting plasmids pGL3-Basic, PFBDM, p-VD1, and p-VD2 were constructed and preserved in this laboratory, and the PGL3-Ie1-PA vector was previously constructed and maintained in our lab. All schematic diagrams of plasmid construction are shown in Supplementary Figure S1. Information on the construction of the recombinant vectors PFast-ie1-NS2-eGFP, pIB-NS1-mCherry, and pGL3-p10-NS2 is provided in the Supplementary Materials.

BmN cells were cultured in TC-100 supplemented with 10% fetal bovine serum (FBS) (Gibco, Thornton, NSW, Australia) and 1% Penicillin-Streptomycin (Gibco, Grand Island, NY, USA), maintained in a 27 °C incubator.

### 2.3. Silk Worm Rearing and Baculovirus Infection

*Bombyx mori* larvae (306 strain) were fed with mulberry leaves at 25 °C and 60% humidity up to the fifth instar and injected with the recombinant baculovirus.

Following the manufacturer’s instructions for the Bac-to-Bac Baculovirus ExpressionSystem (Invitrogen, Carlsbad, CA, USA), we constructed two Bacmids by means of the transposition of the above two plasmids with DH10BmBac^TM^ *E. coli* competent cells. The primers used are listed in Supplementary Materials Table S2. Bacmid DNA, isolated from amplified bacterial colonies, was used to transfect BmN cells to generate recombinant BmNPV, which was named rBmNPV-P5.5-NS2-eGFP. The second passage of the virus, collected from BmN cells, was used for silkworm infection.

### 2.4. Co-IP

After the silkworm larvae were infected with recombinant baculovirus for 5 days, we dissected the diseased silkworms to obtain the midgut, weighed 100–200 mg of silkworm midgut sample, and cut it into small pieces. We then added 1 mL of inhibitor-containing lysate and let it stand for 30 min, centrifuged it at 12,000× *g* at 4 °C for 5 min, and extracted the supernatant. We added the prepared 1 mL silkworm intestinal lysate to the magnetic beads (using the protein A + G magnetic bead method, Beyotime, Shanghai, China) that were bound to the antibody (Abmart, Shanghai, China) and incubated them overnight under a 4 °C rotation. After the incubation was complete, we placed the samples on a magnetic rack to separate for 10 s and removed the supernatant. We then added 0.5 mL of inhibitor-containing lysate, gently pipetted the resuspended magnetic beads, placed them on a magnetic frame to separate for 10 s, removed the supernatant, repeated the washing, and retained the washing solution, establishing that the washing solution OD280 was not greater than 0.05 when the washing stopped, generally after 5 times. Finally, it was separated on a magnetic frame for 10 s, and after removing the supernatant, 150 μL of 1× protein loading buffer was added to resuspend the mixture, which was placed in a metal bath at 95 °C for 10 min. After cooling to room temperature, it was placed on a magnetic rack for magnetic separation for 10 s, and the supernatant was collected for WB or mass spectrometry detection.

### 2.5. Mass Spectrometry

We diluted the final SDS concentration of the protein sample to less than 0.1% using 0.5 M TEAB and added a Trypsin enzyme solution at a ratio of 1 (protein): 20 (enzyme), after which we gently centrifuged the mixture after vortex shaking and carried out enzymatic lysis at 37 °C for 4 h. The digested peptide solution was desalinated and then frozen and drained. After draining, the peptide sample was remixed with a mobile phase and centrifuged at 20,000× *g* for 10 min, and the supernatant was taken for on-the-machine detection. The UltiMate 3000 UHPLC (Thermo Fisher Scientific, Waltham, MA, USA) was used for separation. The detection of liquid-phase isolated peptides was carried out using the DDA (Data-Dependent Acquisition) mode of the tandem mass spectrometer Q-Exactive HF X. The selected databases are the NCBI Silkworm Database (https://www.ncbi.nlm.nih.gov/genome/?term=Bombyx+mori, accessed on 11 May 2024) and the Southwest University Silkdb3.0 Database (https://silkdb.bioinfotoolkits.net, accessed on 11 May 2024).

We carried out a GO (Gene Ontology) functional annotation analysis of all the identified proteins, and the results include two parts: protein2go and go2protein. Protein2go is a list of IDs and all corresponding GO functions for each protein. Go2protein provides the GO entries involved in the three ontologies (cellular component, biological process, molecular function). Go2protein lists the IDs and numbers of all the corresponding proteins and presents a statistical chart. GO entries without corresponding proteins are excluded. The analysis blasts the identified proteins against the KOG (Eukaryotic Orthologous Groups) database, predicting the possible functions of these proteins, and performing functional classification statistical analysis.

## 3. Results

### 3.1. Evolutionary Analysis of NS2 in Invertebrate ssDNA Viruses

**Figure 1:**
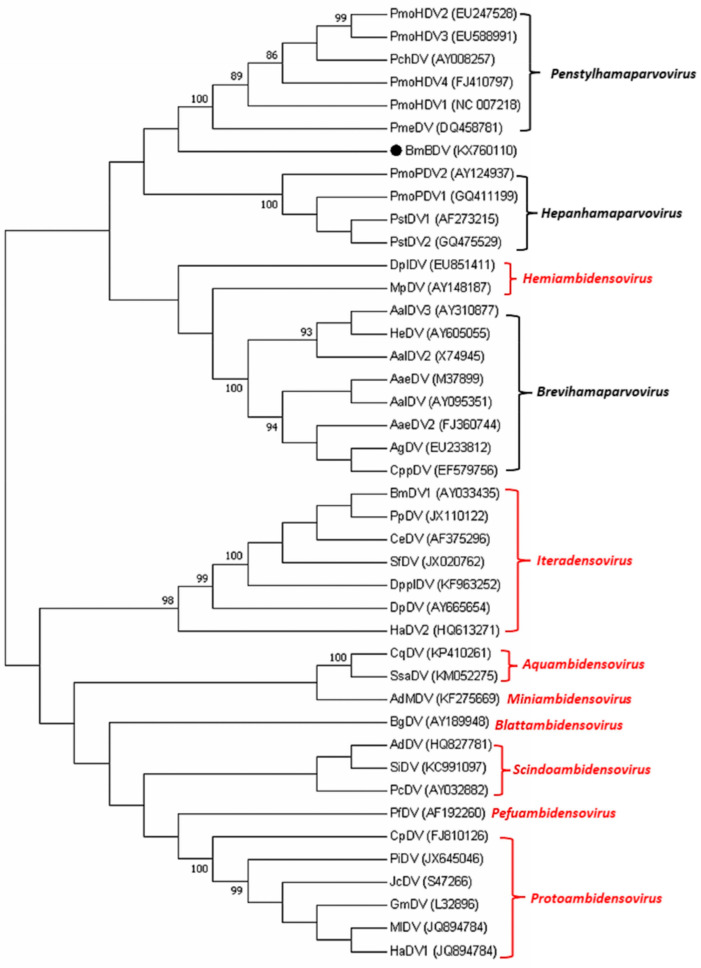
Phylogenetic analysis of invertebrate ssDNA viruses’ NS2. Note: for the evolutionary analysis using MEGA7, we inferred the evolutionary history using adjacency. The optimal tree with a total branch length of 17.05648256 is shown. The percentage of repeat trees where the relevant taxa clustered together in the guided test (2000 replicates) is shown next to the branch. The evolutionary distance was calculated using the Poisson correction method, measured as the number of amino acid substitutions at each site. The analysis contained 42 amino acid sequences. We removed all locations containing blank and missing data. There are a total of 113 locations in the final dataset. At the side of the evolutionary tree, the genus of the virus is marked: red indicates the genus of viruses within the subfamily *Densovirinae*, and black indicates the genus of viruses within the subfamily *Hamaparvovirinae*.

We performed multiple sequence alignments and phylogenetic analysis on all the NS2s of the invertebrate parvoviruses and BmBDV obtained from GenBank using MEGA 7.0 software. The results are depicted in Figure 1. The clustering of the invertebrate parvovirus NS2 proteins closely matches the most current taxonomy. The NS2 protein of BmBDV is evolutionarily proximate to the *Hamaparvovirinae* subfamily, and within the phylogenetic tree’, we also identified that Myzus persicae densovirus (MpDV) and Dysaphis plantaginea densovirus (DplDV), which currently belong to the *Hemiambidensovirus* genus of the *Densovirinae* subfamily, have a closer evolutionary relationship with the *Brevihamaparvovirus* genus of the *Hamaparvovirinae* subfamily.

### 3.2. Intracellular Localization and Nuclear Localization Signal Analysis of BmBDV Non-Structural Protein NS2

**Figure 2:**
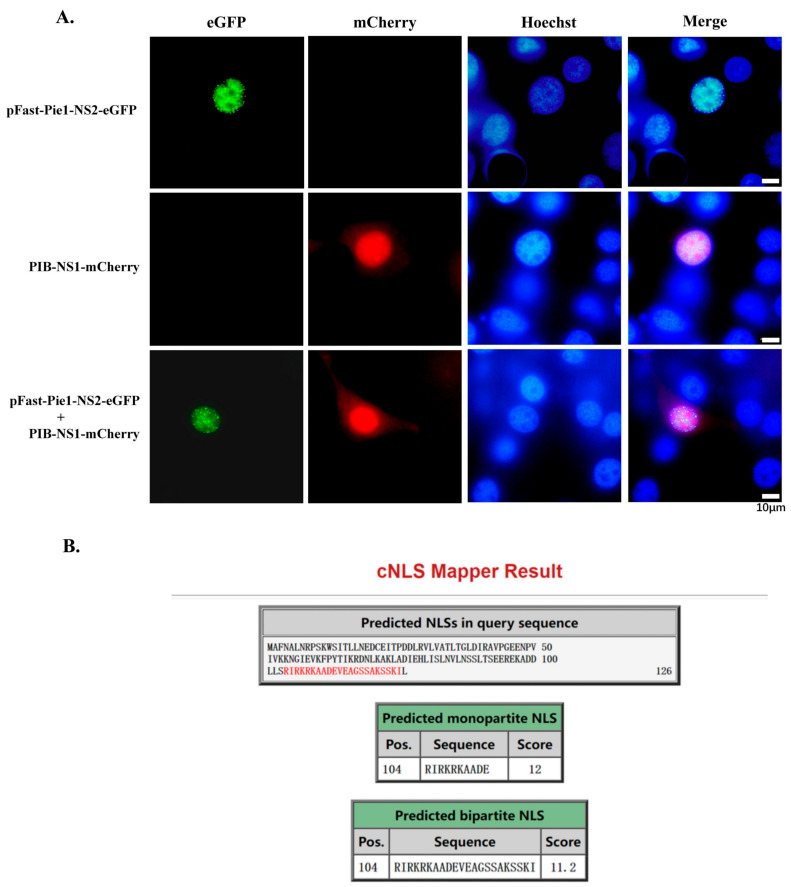
Subcellular localization of BmBDV NS2 and prediction result for NLS. (**A**) Subcellular localization of NS1-mCherry and NS2-eGFP. (Bar: 10 μm). (**B**) Prediction results of BmBDV NS2 NLS from NLS mapper website.

In the study of the function of ssDNA virus proteins in invertebrates, there have been relatively many reports on NS1 proteins, and the protein function is currently relatively clear. Most viruses encode the NS2 protein simultaneously, which is either expressed by the transcript of NS1 through the ribosome leak scanning mechanism or expressed separately from NS1 in their respective transcripts. The encoding regions of these two proteins often have significant overlap. To investigate whether the intracellular localization of NS1 and NS2 can provide insights into the function of NS2, we constructed an NS1-mCherry expression vector and an NS2-eGFP expression vector, which were co-transfected into BmN cells to observe the co-localization of NS1 and NS2. As shown in Figure 2A, both NS1 and NS2 are localized to the nucleus, but there is no significant co-localization between them. The NS1 protein is predominantly aggregated in the nucleolus, which may be associated with its crucial role in viral genome replication, whereas NS2 is located in other parts of the nucleus, excluding the nucleolus.

Considering the characteristics of NS2 localization in the nucleus, an analysis of NS2 using the NLS mapper website, as illustrated in Figure 2B, revealed that NS2 contains a monopartite NLS with a score of 12 and a bipartite NLS with a score of 11.2.

### 3.3. Analysis of the Types of and Key Amino Acids of the NS2 Protein’s NLS

**Figure 3:**
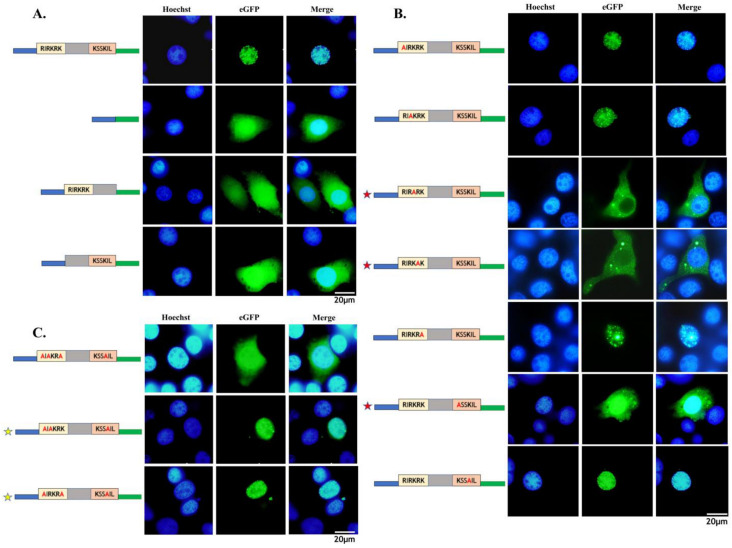
Evaluation of the functional NLS elements and identification of critical amino acids. (**A**) The BmBDV NS2 gene containing a bipartite NLS. (**B**) Single-mutation analysis of basic amino acids to identify key amino acids of the bipartite NLS. The red star marks the critical mutation of basic a.a. at the 107, 108, and 121 positions. The subcellular localization of NS2 will be restricted within the cytoplasm after the mutagenesis of these three amino acids. (**C**) Retaining only the key amino acids does not restore the nuclear localization of NS2. The yellow stars mark the critical recovery of key amino acids for rescuing the nuclear localization of NS2. Boxes of different colors mark the two potential functional areas of NLS, with green lines indicating EGFP. (Bar: 20 μm).

Based on the NLS prediction analysis results, we assume that the NLS of NS2 is a bipartite type. After constructing the NS2-eGFP fusion expression vector (pFast-Pie1-NS2-eGFP), we performed deletion mutations on the potential NLS functional domains. The recombinant vector, after the deletion of the NLS domain, was transfected into BmN cells, resulting in the dispersion of NS2-eGFP throughout the cell, indicating that the NLS located at the C-terminus is functional and consistent with the software predictions. We further truncated mutations of the two basic amino-acid-rich regions within the NLS, demonstrating that the NLS of NS2 is a bipartite type (Figure 3A).

Subsequently, by performing single-point mutations of the basic amino acids in these two regions to alanine (A), we identified three key amino acids that directly affect the localization of NS2: lysine (K) at position 107, arginine (R) at position 108, and lysine (K) at position 121. When attempting to retain only these key amino acids and mutate the other basic amino acids to verify their function, we found that retaining only these three key amino acids did not allow NS2 to enter the nucleus (Figure 3B). Therefore, by retaining the key amino acids and gradually restoring other basic amino acids, we discovered that the realization of NS2’s NLS function requires lysine (K) at position 107, arginine (R) at position 108, and lysine (K) at position 121, as well as either arginine (R) at position 106 or lysine (K) at position 109 (Figure 3C). From this, it can be concluded that the combination rule for the functional NLS of BmBDV is (R)KR(K)X10-12K3/5, where X represents any amino acid, and at least one of the basic amino acids in parentheses must be retained.

### 3.4. Prediction of NLS in NS2 Protein of Invertebrate ssDNA Viruses

The predicted NLSs in the NS2 proteins of invertebrate ssDNA viruses are summarized in Table 1. The prediction results indicate that there are regions that are rich in basic amino acids within the NS2 protein sequences of invertebrate ssDNA viruses. Moreover, there is conservation in the positioning of these basic amino acid-rich regions among different viruses within the same genus. Based on this information, we speculate that even though the NS2 protein sequences may have low conservation, there might be a certain level of functional conservation across various genera of the virus. Additionally, the NLS of the NS2 in invertebrate ssDNA viruses may have unique combinatorial patterns, and some viruses do not conform to the classic patterns, resulting in lower scores (<8.0) in software predictions.

### 3.5. Impact of BmBDV NS2 on the Promoter Activity of Viral Proteins

**Figure 4:**
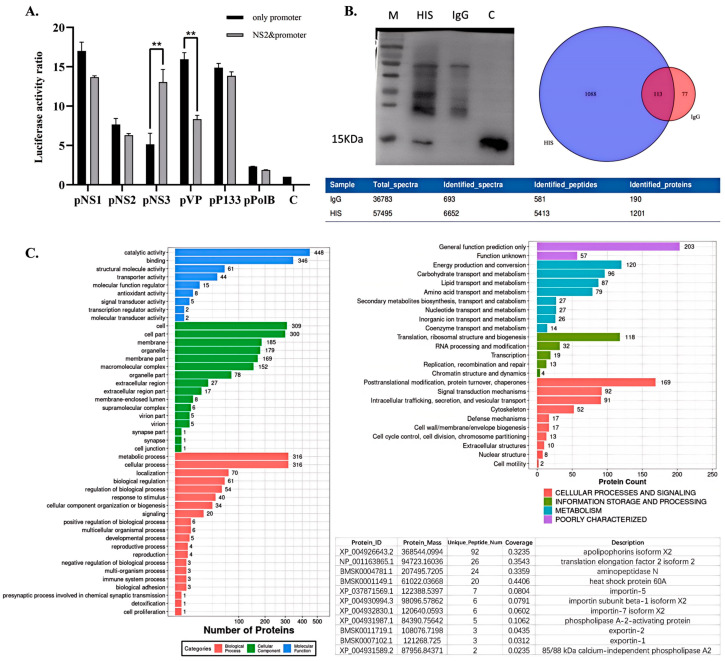
Analysis of the effect of the NS2 protein on the activity of various viral protein promoters of BmBDV and the potential interacting host proteins of NS2. (**A**) The luciferase reporter plasmids contain different viral proteins’ promoter elements. An NS2 overexpression plasmid was constructed by replacing luciferase ORF with NS2 ORF controlled by a P10 promoter. BmN cells were transfected with the promoters individually or co-transfected with the NS2 overexpression plasmid. The luciferase activity was observed. The black boxes show the original promoter activity, and the gray boxes show promoter activities after co-transfection with NS2. (*n* = 3). Error bars denote standard deviation. **, *p* < 0.05. (**B**) Western blot detection of the NS2 Co-IP results and NS2 expression in the *Bombyx mori* midgut. The mass spectrometry peptide identification results were compared between IgG and His samples. A Wayne diagram showed the number of different proteins. (**C**) GO and KOG analysis of specific proteins in HIS experimental group.

Using the dual-luciferase system, we investigated the transcriptional influence of NS2 on the promoters of BmBDV proteins. Our results indicate (as shown in Figure 4A) that, 48 h post-co-transfection, the overexpression of the NS2 protein positively regulates the promoter activity of the non-structural protein NS3, denoted as P10, while it may have an inhibitory effect on the promoter of the structural protein VP, known as P21. This suggests that the expression of NS2 may play a role in regulating the expression levels and timing of various proteins during the viral replication process.

### 3.6. Analysis of Host Proteins That Interact with NS2 Using Co-IP and LC-MS/MS

We constructed a recombinant baculovirus containing the NS2-His expression cassette, which was then used to infect silkworms. After the silkworms exhibited disease symptoms, their midguts were collected for co-immunoprecipitation (Co-IP) protein sample preparation. Western blot detection was performed using mouse anti-HIS as the experimental group (Figure 4B). The isolated protein complexes were subjected to liquid chromatography–tandem mass spectrometry (LC-MS/MS) analysis for identification. In the HIS experimental group, 1201 proteins and 5413 peptides were identified, while 190 proteins and 581 peptides were identified in the IgG control group. We constructed a Venn diagram of the differential protein analyses of both datasets, excluding non-specific proteins that were common to both the experimental and IgG control groups, leaving 1088 specific proteins (Figure 4B). The specificity of the His antibody experimental group was further analyzed using GO analysis and KOG annotation, as shown in Figure 4C. We sorted the 1088 proteins that were obtained from the mass spectrometry analysis based on the number of unique peptide matches and selected proteins of interest based on their protein coverage, considering results with more than two unique peptide matches to be reliable. The specific proteins included those related to nuclear import and export, such as Importin-5, Importin subunit beta-1 isoform X2, Importin-7 isoform X2, Exportin-2, Exportin-1, and Protein SEC13 homolog. These results further support the nuclear localization properties of NS2 and suggest its potential functions in the viral replication process.

## 4. Discussion

The present investigation has delineated that the non-structural protein NS2 of BmBDV exhibits nuclear localization, a characteristic with profound implications for elucidating its role within the viral life cycle. The nuclear translocation of this protein is governed by a functional bipartite NLS situated at the C-terminus, which diverges from the composition of classical NLSs, exhibiting unique features in its basic amino acid arrangement. This finding is significant as it highlights the potential for non-classical NLSs to play crucial roles in viral protein trafficking, a concept that has been increasingly recognized in recent studies of viral nuclear import mechanisms.

Our findings are in line with previous research on parvoviruses, which have established the importance of NLSs in the nuclear transport of viral proteins and genomic materials [13]. The phylogenetic analysis presented in this report supports the close evolutionary relationship of invertebrate ssDNA viruses’ NS2 proteins, despite the low sequence conservation. The presence of a functional bipartite NLS in BmBDV NS2, as identified in this study, is consistent with the evolutionary conservation of NLSs among invertebrate ssDNA viruses. This is in agreement with the bioinformatical analysis of penaeid densoviruses, which also revealed functional NLSs in these viruses [8]. This conservation suggests a common mechanism for nuclear import, which is crucial for viral replication and assembly.

We confirmed that the combined form of a functional bipartite NLS in BmBDV NS2 protein is (R)KR(K)X10-12K3/5, where X represents any amino acid, and at least one of the basic amino acids in parentheses must be retained. Unlike the classic bipartite NLS with a general sequence motif of R/K(X)10-12RRKK, the NLS of BmBDV NS2 protein has its own compositional characteristics.

We also attempted to summarize the potential NLS domains in the NS2 protein of all invertebrate ssDNA viruses in Table 1. This is also the first NLS analysis of the NS2 protein of all invertebrate ssDNA viruses after ICTV reclassified the *Parvoviridae* family [14]. Although the results predicted by the software indicate that they are either in the form of mono- or bipartite, most of them do not conform to the classic NLS combined form. For instance, the software prediction showed that the NS2 protein of JcDV contains a potential bipartite NLS (Table 1), but we validated through deletion and point mutation of key amino acids that the key functional amino acids are only located in the three adjacent basic amino acids near the C-terminus, indicating that the nuclear localization function of JcDV’s NS2 is controlled by a monopartite NLS (Supplementary Figure S2). Further experiments are still needed to confirm their true functional amino acids.

The role of NS2 in BmBDV appears to be multifaceted, as it not only contains a functional NLS but also influences the viral promoter activity, as evidenced by the dual-luciferase reporter system. This is reminiscent of the role of NS2 in the minute virus of mice (MVM), where NS2 is critical for DNA replication and the production of progeny single-strand DNA (ssDNA) [15]. The dependence of MVM replication on NS2 accumulation highlights the potential importance of NS2 in the replication of BmBDV and other parvoviruses. Studies have shown that BmBDV NS2 is closely related to adenovirus death protein (ADP), which is essential for cell lysis and virus release [16]. ADP is expressed early and amplifies significantly in the later stages of infection, while NS3 may function in the lysis of cells to release the virus. In the late stages of viral replication, when VP reaches a certain level of expression, the NS2 protein may regulate virus assembly and cell lysis for virus release by repressing the transcription of VP and promoting the expression of NS3. However, these hypotheses still require further in-depth research and validation.

Furthermore, the interaction of BmBDV NS2 with host proteins, as attempted in this study, mirrors the interaction of MVM NS2 with the nuclear export protein Crm1 [15]. Such interactions may be crucial for the nuclear egress of progeny virions, as suggested by a study focusing on MVM [17]. Recent studies have demonstrated that NLS in non-structural proteins not only aids in nuclear import but also plays essential roles in regulating viral replication and modulating host immune responses [17,18]. For instance, the non-structural protein NS2 of various viruses has been shown to contain functional NLSs that are critical for viral replication and pathogenesis [13]. Inspired by these research findings, we conducted preliminary experiments to detect the interaction between the NS2 protein of JcDV and the host importin α of Sf9 cells. We found that the NS2 protein has significant interactions with importin α1 and importin α7. These results will be further refined in future work.

In conclusion, the findings of this study expand our understanding of the role of NS2 in BmBDV and its conservation among invertebrate ssDNA viruses. The functional NLS of NS2 and its impact on viral promoter activity underscore the importance of nuclear transport in the viral life cycle. Future research should focus on the detailed mechanisms of NS2-mediated nuclear transport and its interaction with host factors, which could contribute to the development of therapeutic vaccines and antiviral medicines, as suggested by Liu et al. [13].

## Figures and Tables

**Table 1:** NLS prediction of invertebrate ssDNA viruses’ NS2 proteins.

Subfamily	Genus	Species	Virus	Position (Full Length)	NLS Candidate Sequence
*Hamaparvovirinae*	*Penstylhamaparvovirus*	*Decapod penstylhamaparvovirus 1*	PstDV1 (AF273215)	310–320 (363)	PKLKRLRYLLE
			PstDV2 (GQ475529)	310–320 (363)	PKLKRLRYLLE
			PmoPDV1 (GQ411199)	310–320 (363)	PKLKRLRYLLE
			PmoPDV2 (AY124937)	173–183 (226)	PKLKRLRYLLK
	*Hepanhamaparvovirus*	*Decapod hepanhamaparvovirus 1*	PmoHDV1 (NC_007218)	280–290 (428)	DEPANKKRKFC
			PmoHDV2 (EU247528)	201–211 (349)	EEPASKKRKFS
			PmoHDV3 (EU588991)	196–206 (344)	EEPASKKRKFS
			PmoHDV4 (FJ410797)	278–288 (426)	DEPASKKRKFS
			PchDV (AY008257)	200–210 (348)	DEPANKKRKFS
			PmeDV (DQ458781)	177–202 (340)	KGKDKRDQQKTEKKDDEPPKKKTKFS
	*Brevihamaparvovirus*	*Dipteran brevihamaparvovirus 1*	AgDV (EU233812)	284–306 (383)	KTTLKRTGDTSPQPGPSKRRVSS (ls.)
			AaeDV1 (M37899)	263–285 (362)	KTTLKRTGDTSPQPGPSKRRVSS (ls.)
			AaeDV2 (FJ360744)	263–285 (362)	KTTLKRTGDTSPQQGPSKRRVSS (ls.)
			CppDV (EF579756)	264–286 (363)	KTTLKRTGDTSPQPGPSKRRVSS (ls.)
		*Dipteran brevihamaparvovirus 2*	AalDV (AY095351)	264–286 (363)	KTTLKRTGDTSPQPGPSKRRVSS (ls.)
			AalDV2 (X74945)	264–286 (363)	KITAKRTGDISPQQGPSKRRALS
			AalDV3 (AY310877)	264–286 (363)	KTTAKRTGDISPQPGPSKRRALS
			HeDV (AY605055)	264–286 (363)	KITAKRTGDISPQQGPSKRRALS
*Densovirinae*	*Iteradensovirus*	*Lepidopteran iteradensovirus 1*	BmDV1 (AY033435)	271–295 (451)	RHINTTRKRKSITTSKGVLTKKKSLK
		*Lepidopteran iteradensovirus 2*	CeDV (AF375296)	271–295 (452)	KHTNTTRKRKSTMMSTGKFMKKKLL
			DppIDV (KF963252)	271–295 (451)	RYTSTTQKRKSTTTHKGKFTKKKIL (ls.)
			SfDV (JX020762)	272–296 (452)	RHTNTTQKRKSTTTHKGKFTKKKLL (ls.)
		*Lepidopteran iteradensovirus 3*	HaDV2 (HQ613271)	252–277 (419)	QDDDTSSKETESSGTSEPSKKKQKLS
		*Lepidopteran iteradensovirus 4*	PpDV (JX110122)	275–299 (455)	GYTSTTQKRKSSTTLKGQFTKKKIL
		*Lepidopteran iteradensovirus 5*	DpDV (AY665654)	278–295 (453)	MTPKSKKSTKLEKAGRSKGQ
	*Miniambidensovirus*	*Orthopteran miniambidensovirus 1*	AdMDV (KF275669)	126–144 (222)	TRNKRGRCTYKWVPPKLKS
	*Aquambidensovirus*	*Decapod aquambidensovirus 1*	CqDV (KP410261)	176–185 (305)	AGATGTKRRK
		*Asteroid aquambidensovirus 1*	SsaDV (KM052275)	163–172 (294)	AGATGGKRRK
	*Scindoambidensovirus*	*Orthopteran scindoambidensovirus 1*	AdDV (HQ827781)	274–282 (286)	RPSPKKQKY
		*Hymenopteran scindoambidensovirus 1*	SiDV (KC991097)	272–283 (288)	RKDPNLSGYRRRYIPY
		*Hemipteran scindoambidensovirus 1*	PcDV (AY032882)	263–272 (273)	LKKSSKRYRP
	*Protoambidensovirus*	*Lepidopteran protoambidensovirus 1*	GmDV (NC_004286)	252–274 (274)	FMKRKPIRQGNSHTYGKKQRRY
			JcDV (KC883978)	251–275 (275)	FMKRKPIRQGNSHTYGKRQKRY
			MlDV (AY461507)	251–275 (275)	FMKRKPIRQGNSHTYGKRQRRY
			HaDV1 (JQ894784)	251–275 (275)	FMKRKPIRQANSHTYGKRQRRY
			PiDV (JX645046)	251–275 (275)	FMKRKPIRQGNSHTYGKRQKRY
		*Dipteran protoambidensovirus 1*	CpDV (FJ810126)	252–273 (274)	FMAKKPNRQENMQLLNRKRKRY
	*Hemiambidensovirus*	*Hemipteran hemiambidensovirus 1*	DpIDV (EU851411)	33–55 (351)	RKKHNSVLGFSLDEPRQKKQKPQ
		*Hemipteran hemiambidensovirus 2*	MpDV (AY148187)	193–221 (249)	RRRRIRKNVRVIQLSERDWSRIFQYLCSS
	*Pefuambidensovirus*	*Blattodean pefuambidensovirus 1*	PfDV (AF192260)	141–165 (265)	KSSKKRQLKLSVAERLENVKRLAQS
	*Blattambidensovirus*	*Blattella germanica densovirus 1*	BgDV (AY189948)	138–163 (262)	RTSLKRKKTLDDCFESLQPSKRSHDE
*Bidnaviridae*	*Bidensovirus*	*Bombyx mori bidensovirus*	BmBDVVD1: KX760110	104–126 (126)	RIRKRKAADEVEAGSSAKSSKIL

(Note: All viruses were classified according to the latest ICTV classification. The names of all viruses are followed by their sequence number in the GenBank (NCBI). The candidate NLSs are listed, and basic amino acids are colored in red. The position column represents the position of the protein sequence in which the predicted NLS is located and the total length of the protein sequence. Finally, l.s. indicates that the prediction score from the NLS mapper is lower than 8.0, low score).

## Data Availability

The original contributions presented in the study are included in the article. Requests for reagents developed in this work such as cell lines and plasmids as well as raw images can be directed to the corresponding author.

## Supplementary Materials

The following supporting information can be downloaded at https://www.mdpi.com/article/10.3390/v17010071. Figure S1: Schematic diagram of plasmid construction [19]; Figure S2: Evaluation of the functional NLS elements of JcDV NS2 and identification of critical amino acids; Table S1: The primers used in the Site direct mutagenesis of NS2 NLS; Table S2: The primers used in the Dual Luciferase Reporter Assay; Table S3: The primers used in Bac-to-Bac system.
