# Supplementary material for: Conserved Nuclear Localization Signal in NS2 Protein of Bombyx Mori Bidensovirus: A Potential Invertebrate ssDNA Virus Trait
Source: Viruses. 2025 Jan 6;17(1):71. doi: 10.3390/v17010071 (PMC11768917; doi:10.3390/v17010071)
Supplement: Supplementary file 1 [file viruses-17-00071-s001.zip › viruses-3402272-supplementary.pdf]

Supplementary materials

Conserved Nuclear Localization Signal in NS2 Protein of Bombyx Mori Bidensovirus: A Potential Invertebrate ssDNA Virus Trait

Qian Yu*, Jiaxin Yan, Ying Chen, Jinfeng Zhang, Qi Tang, Feifei Zhu, Lindan Sun, Shangshang Ma, Xiaoyong Liu, Keping Chen and Qin Yao

School of Life Sciences, Jiangsu University, Zhenjiang 212013, China; qianyu@ujs.edu.cn (Q.Y.); yanjiaxin9899@163.com (J.Y.); 3224501003@stmail.ujs.edu.cn (Y.C.); 15689321131@163.com (J.Z); tangqi1224@163.com; (Q.T.); feifzhu@ujs.edu.cn (F.Z.); sunlindan@ujs.edu.cn (L. S.)；mashang@ujs.edu.cn (S.M.); liuxiaoyong@ujs.edu.cn (X.L.); kpchen@ujs.edu.cn (K.C.); yaoqin@ujs.edu.cn (Q.Y.)

* Correspondence: qianyu@ujs.edu.cn (Q.Y.); Tel.: +86-15050853270 (Q.Y.)

1. Plasmid construction

The recombinant plasmids used in the project were constructed into pFastBacDual cloning vector (Invitrogen, Carlsbad, CA, USA) which was described in the previous work [19].

pFastHTB-ie1-NS2-eGFP: For NLS detection

pGL3-P10-NS2: For Dual Luciferase Analysis

pFBMD-NS2: For recombinant baculovirus construction.

pFast-ie1-NS2-eGFP, pIB-NS1-mCherry, pGL3-p10-NS2 recombinant vectors were constructed using bi-enzyme digestion. Deletion mutations and multi-/single-point mutations were performed on the key alkaline amino acids of the functional domain of the predicted NS2 protein by means of the homologous recombination method (regarding primers, see Table S1). Cultured BmN cells were passed to a 24-well plate with pre-placed cell crawling tablets at a density of around 80%. After 4 h of constant-temperature culture, we washed the cells twice with PBS and replaced with an antibiotic- and serum-free medium. We then took two sets of ep tubes and added 50 μL of antibiotic- and serum-free medium to each group: 1.5－2 μg of DNA to group A and 2 μL cellfectin to group B (Cellfectin Reagent II, Gibco, Jenks, OK, USA). After light suction mixing, we added A to B and placed the mixture at room temperature for 30 min, during which the tube wall promoted the binding of liposomes to the DNA. A pipetting and transfecting mix was added to the cell wells, placed in the incubator for 6 h, and gently shaken on the cell plate during this period. After 6 h, the medium was replaced with a normal TC-100 medium and placed in a constant-temperature incubator to continue the culture. After 48hr incubation, the cells were used to downstream analysis.

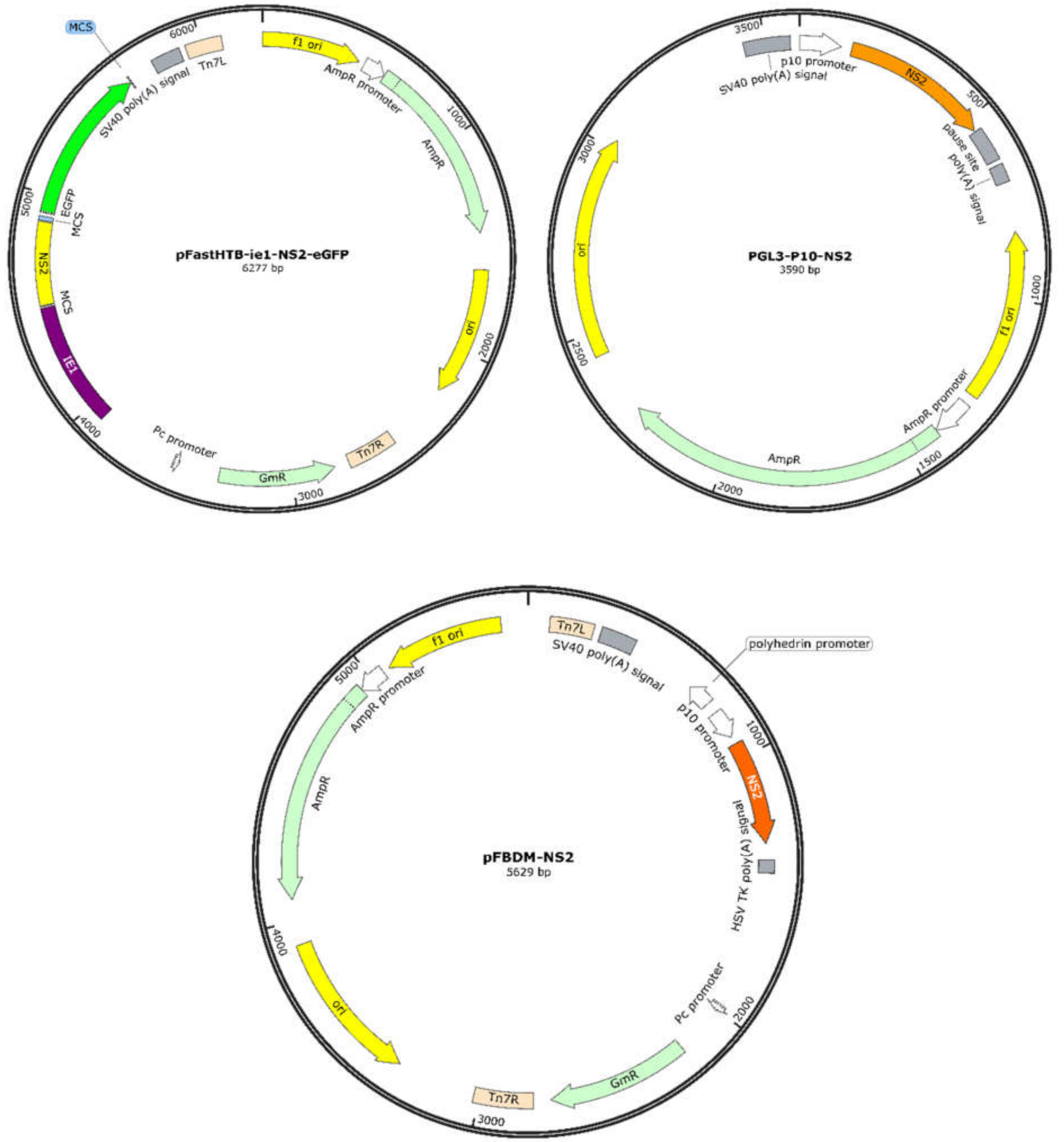

Supplementary Figure S1. Schematic diagram of plasmid construction.

2. Primer sequences:

Table S1: The primers used in the Site direct mutagenesis of NS2 NLS

Primers	Mutation Site	Sequences (5'→3')
**pFast-ie1-M5-eGFP F**	M5:^{104}RIRKRKAADEVEAGSSA^{121}ASSKIL^{126}	aagtgcagcgagctctaagattctgctgcagatg
**pFast-ie1-M5-eGFP R**		tagagctcgctgcacttgagccagcttcgact
**pFast-ie1-M6-eGFP F**	M6:^{104}RIRKRKAADEVEAGSSA^{121}KSSAIL^{126}	agagctctgcgattctgctgcagatggtgagc
**pFast-ie1-M6-eGFP R**		cagaatcgcagagctctttgcacttgagccag
**pFast-ie1-M7-eGFP F**	M7: ^{104}AIRKRKAADEVEAGSSA^{121}KSSKIL^{126}	tcctctctgcaatcaggaaacggaaagcagcag
**pFast-ie1-M7-eGFP R**		tcctgattgcagagaggagatcgtcagcctttt
**pFast-ie1-M9-eGFP F**	M9: ^{104}RIAKRKAADEVEAGSSA^{121}KSSKIL^{126}	ctagaatcgcgaaacggaaagcagcagatgaag
**pFast-ie1-M9-eGFP R**		tccgtttcgcgattctagagaggagatcgtcagcc
**pFast-ie1-M10-eGFP F**	M10: ^{104}RIRARKAADEVEAGSSA^{121}KSSKIL^{126}	agaatcagggcacggaaagcagcagatgaagtc
**pFast-ie1-M10-eGFP R**		tttccgtgccctgattctagagaggagatcgtca
**pFast-ie1-M11-eGFP F**	M11: ^{104}RIRKAKAADEVEAGSSA^{121}KSSKIL^{126}	tcaggaaagcgaaagcagcagatgaagtcgaag
**pFast-ie1-M11-eGFP R**		ctgctttcgctttcctgattctagagaggagatcg
**pFast-ie1-M12-eGFP F**	M12: ^{104}RIRKRAAADEVEAGSSA^{121}KSSKIL^{126}	agaatcaggaaacgggcagcagcagatgaagt
**pFast-ie1-M12-eGFP R**		acttcatctgctgctgcccgtttcctgattct
**pFast-ie1-M13-eGFP F**	M13: ^{104}AIAKRAAADEVEAGSSA^{121}KSSAIL^{126}	gcaatcgcgaaacgggcagcagcagatgaagtcgaagct
**pFast-ie1-M13-eGFP R**		atctgctgctgcccgtttcgcgattgcagagaggagatcgtc
**pFast-ie1-M14-eGFP F**	M14: ^{104}AIAKRAAADEVEAGSSA^{121}KSSKIL^{126}	gcaatcgcgaaacgggcagcagcagatgaagtcgaagct
**pFast-ie1-M14-eGFP R**		atctgctgctgcccgtttcgcgattgcagagaggagatcgtc
**pFast-ie1-M15-eGFP F**	M15: ^{104}AIAKRKAADEVEAGSSA^{121}KSSKIL^{126}	ctctctgcaatcgcgaaacggaaagcagcagat
**pFast-ie1-M15-eGFP R**		tttcgcgattgcagagaggagatcgtcagcctttt
**pFast-ie1-M16-eGFP F**	M16: ^{104}AIRKRAAADEVEAGSSA^{121}KSSKIL^{126}	tcctctctgcaatcaggaaacgggcagcagcag
**pFast-ie1-M16-eGFP R**		tcctgattgcagagaggagatcgtcagcctttt

Table S2: The primers used in the Dual Luciferase Reporter Assay

Primers	Sequences (5’→3’)
P10-NS3 *Kpn* I F	ggggtacctactcgccatctctcc
P10-NS3 *Hin*d III R	cccaagcttcggttaaacgtaacatattg
p21-VP *Kpn* I F	ggggtacctctttttaaatggtaatataaagactgg
P21-VP *Xho* I R	ccctcgaggagccaagtactctaccc
p89-P133 *Kpn* I F	ggggtacctgtgtgtatactggggcg
p89-P133 *Hin*d III R	cccaagcttgcgcaaaatacgtataacctc
p97-PolB *Kpn* I F	ggggtaccgtgtgtgtatactggggcg
p97-PolB *Hin*d III R	ccctcgagcatcttgaagttacagaatggc
NS2 *Xho* I F	ccctcgagatggcattcaacgct
NS2 *Kpn* I R	ggggtaccctacagaatcttagagctc
P10+NS2 *Eco*R I F	ggaattcgacctttaattcaacccaac

Table S3: The primers used in Bac-to-Bac system

Primers	Sequences (5’→3’)
ie1-eGFP *Xho* I F	ccctcgaggtaggttattgataaaatgaacgg
ie1-eGFP *Hin*d III R	cccaagcttttacttgtacagctcgtcca
p5.5+NS2+His *Xho* I F	ccctcgagatttatactttaagcccatacaaata
p5.5+NS2+His *Kpn* I R	ggggtacctcagtggtggtggtggtggtgcagaatcttagagctctttg

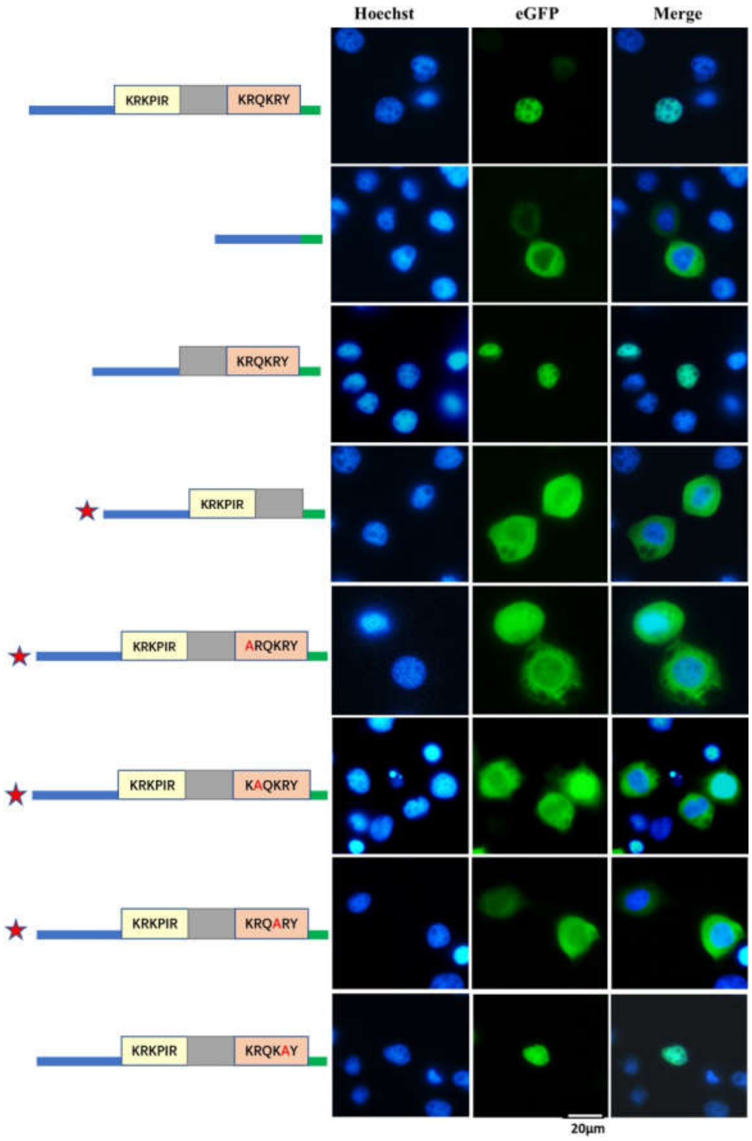

Supplementary Figure S2. Evaluation of the functional NLS elements of JcDV NS2 and identification of critical amino acids. The red star marked the critical mutation or deletion of basic a.a.. The subcellular localization of NS2 will be restricted within the cytoplasm after the mutagenesis of these three amino acids. The red star marked the critical mutation of basic a.a. at 270,271 and 273 position. (Bar: 20μm)
